# Strategic use of levofolinic acid for methotrexate-induced side effects in juvenile idiopathic arthritis: a prospective observational study

**DOI:** 10.1186/s12969-023-00800-3

**Published:** 2023-02-15

**Authors:** Giorgia Martini, Alessandra Meneghel, Michele Fastiggi, Filippo Dell’Apa, Fabio Vittadello, Francesco Zulian

**Affiliations:** Department of Woman and Child Health, Pediatric Rheumatology Unit, University of Padova, 35128 Padua, Italy

**Keywords:** Methotrexate, Side effects, Levofolinic acid, Child, Treatment

## Abstract

**Objective:**

To evaluate the efficacy of levofolinic acid (LVF) administered 48 h before methotrexate (MTX) in reducing gastrointestinal side effects without interference with drug efficacy.

**Methods:**

A prospective observational study was performed including patients with Juvenile Idiopathic Arthritis (JIA) reporting significant gastrointestinal discomfort after MTX despite taking a dose of LVF 48 h after MTX. Patients with anticipatory symptoms were excluded. A LVF supplemental dose was added 48 h before MTX and patients were followed every 3–4 months. At each visit data on gastrointestinal symptoms, disease activity (JADAS, ESR, CRP values) and treatment changes were collected. Friedman test for repeated measures analyzed differences between these variables over time.

**Results:**

Twenty-one patients were recruited and followed for at least 12 months. All patients received MTX subcutaneously (mean 9.54 mg/m2) and LVF 48 h before and after MTX (mean 6.5 mg/dose), 7 received a biological agent too. Complete remission of gastrointestinal side effects was reported in 61.9% of study patients at first visit (T1) and increased over time (85.7%, 95.2%, 85.7% and 100% at T2, T3, T4, T5, respectively). MTX efficacy was maintained as showed by significant reduction of JADAS and CRP (*p* = *0.006 and 0.008*) from T1 to T4 and it was withdrawn for remission in 7/21.

**Conclusions:**

LVF given 48 h before MTX significantly reduced gastrointestinal side effects and did not reduce drug’s efficacy. Our results suggest that this strategy may improve compliance and quality of life in patients with JIA and other rheumatic diseases treated with MTX.

## Background

Methotrexate (MTX) represents an effective and low-cost therapy with relatively safe profile for Juvenile Idiopathic Arthritis (JIA) and JIA-associated uveitis [1, 2]. 

A variable percentage of treated patients, ranging from 11 to 64%, report side effects, mainly gastrointestinal (GI) symptoms, such as nausea and vomiting [3–5]. These symptoms mainly occur during the first 24–36 h after taking the medication and clinical factors associated with higher incidence of adverse effects are older age and longer treatment duration [6]. Some children treated with MTX experience anticipatory nausea that can be triggered by hints like simply seeing the drug packaging or hearing its name, thus suggesting a clear psychological origin [7, 8].

Different methods have been reported to prevent and to manage side effects such as use of anti-emetics, folate supplementation and even switch to much more expensive drugs such as biological agents [9]. These modalities are highly variable from centre to centre, with lack of strong evidence on their efficacy. In our centre all patients receiving MTX are suggested to take a single dose of Levofolinic Acid (LVF) 48 h after MTX but, despite this, some of them report gastrointestinal discomfort. This study was designed to evaluate the efficacy of LVF, administered 48 h before MTX, in reducing MTX-related side effects and to analyze whether this may interfere with drug efficacy.

## Methods

Consecutive patients diagnosed with JIA according to ILAR classification and attending the Pediatric Rheumatology Unit of Department of Woman and Child Health of Padova University and experiencing clinically relevant gastrointestinal complaints on MTX were included in a prospective uncontrolled observational study [10]. All patients were taking LVF 48 h after MTX and despite this reported discomfort. Patients reporting complaints and symptoms before MTX administration, likely of psychological origin, were excluded.

At study entry (T0), patients were suggested to double LVF by taking a supplemental dose 48 h before MTX. Subsequently, patients underwent periodic visits every 3–4 months (T1 to T5) including physical examination, laboratory work-up (complete blood cell counts, ESR, CRP, liver function tests). At each visit the following data were collected: changes in gastrointestinal complaints by a VAS scale defined as: partial remission (symptoms persisting but attenuated with reduction of VAS value at least 30%), complete remission if symptoms disappeared, worsening (increase in VAS value of at least 30%), disease activity (by Juvenile Arthritis Disease Activity Score JADAS calculation, ESR and CRP values) and treatment history (as determined by changes of dosage or frequency of administration of MTX and/or biological agents).

Categorical data were reported in terms of absolute frequencies and percentages. Continuous data were described in terms of mean, SD, median, minimum, and maximum. Friedman test for repeated measures was used to analyze differences between these variables over time.

## Results

Twenty-one patients were recruited, 15 oligoarticular (12 with positive Antinuclear antibodies, ANA), five polyarticular (one Rheumatoid Factor positive) and one enthesitis-related arthritis (ERA) Fifteen were females and the age at onset was 6.9 years on average (median 7 years, range 2–15 years).

At study entry (baseline T0) mean MTX treatment duration was 40 months (median 22, range 2–185 months) and 7 patients were also receiving a biological agent (5 adalimumab, 2 tocilizumab); no patients were on corticosteroids. In all patients, MTX was administered subcutaneously (mean dose 9.54 mg/m^2^/week, range 3.8–14.7 mg/m^2^/week) and associated with LVF 48 h later (mean dose 6.5 mg/week, range 2.5–7.5 mg/week).

Reported side effects after MTX were nausea in 14 patients, vomiting in 5, nausea/vomiting and diarrhea in 2. Seven patients used anti-emetics (ondansetron).

**Fig. 1:**
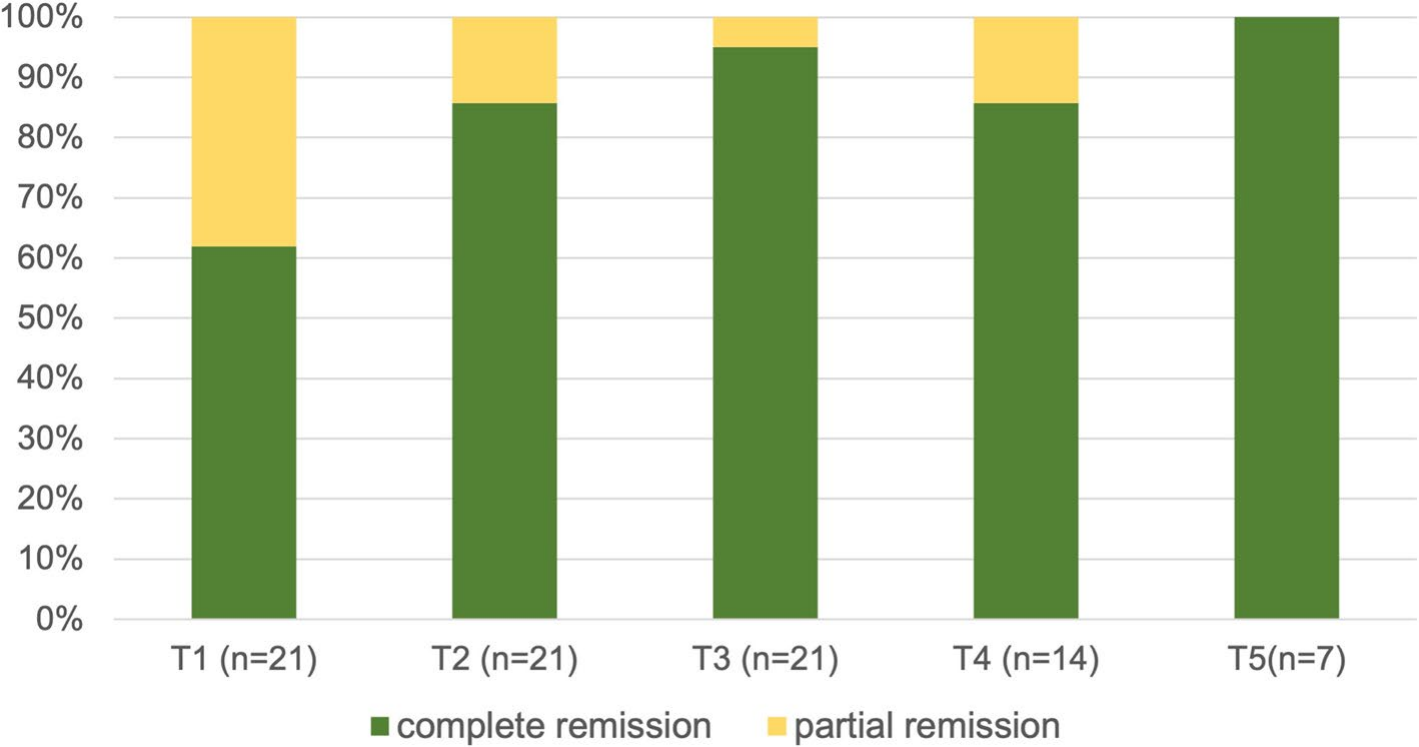
distribution of complete and incomplete remission rates of MTX-related GI adverse effects over the observation period

All patients were followed every 3–4 months for at least 12 months (range 12- 29 months). At first visit (T1) all patients reported improvement of GI complaints with 13/21 (61.9%) experiencing complete remission. The efficacy of LVF pre-MTX persisted over time as a complete remission of GI effects was reported in 18/21 patients (85.7%) at T2, in 20/21 (95%) at T3, in 12/14 (85.7%) at T4 and in 7/7 (100%) at T5, as shown in Fig. 1. No patient reported worsening of the symptoms.

JIA disease activity was not reduced as measured by JADAS, ESR and CRP values during the study period, as reported in Table 1. Friedman test for repeated measures from study start to T4 showed statistically significant differences for JADAS and CRP values (*p* = *0.006 and 0.008,* respectively).

**Table 1:** Values of JADAS, ESR and CRP over the study period. Values are mean and range and statistical significance for differences in repeated measures by Friedman test was considered when < 0.05

	**T0**	**T1**	**T2**	**T3**	**T4**	***p***
**JADAS** Mean (range)	3.77 (0.0–14.0)	2.7 (0.0–8.0)	1.94 (0.0–8.0)	1.36 (0.0–5.0)	1.63 (0.0–5.0)	*0.006*
**ESR** (mm/h) Mean (range)	7.29 (2.0–20.0)	8.5 (2.0–40.0)	4.86 (2.0–22.0)	6.93 (2.0–39.0)	5.93 (2.0–28.0)	*n.s*
**CRP** (mg/L) Mean (range)	3.12 (1.0–11.0)	3.84 (0.5–28.0)	1.64 (0.5–3.0)	2.41 (0.5–12.0)	1.45 (0.5–5.0)	*0.008*

During the first 12 months of study period, MTX doses were stable, then the drug was withdrawn in 7 patients because of disease remission. Two patients started a biological agent (adalimumab) during the study: one for disease relapse after SARS-CoV2 infection, one for persistent disease activity. None of the patients developed hepatic toxicity.

## Discussion

The use of low-dose MTX in the treatment of children with JIA started since the early 90 s, subsequently, it became the most used disease modifying antirheumatic drug (DMARD) [3, 11, 12]. Treatment with MTX widely spread because it is rarely associated with potentially serious side effects such as hepatotoxicity or bone marrow suppression. More commonly, patients experience gastrointestinal adverse effects that can lead to reduced adherence or even to treatment discontinuation.

The prevalence of GI discomfort is not clearly defined. Approximately one third of mothers of JIA patients reported discomfort and 15% vomiting after MTX [7]. In a multicenter trial analyzing the efficacy of oral MTX in 88 children, nausea was described in 28%, but higher rates up to more than 65%, have been reported [4, 6, 7].

Some clinical elements have been associated with development of MTX-induced nausea like older age, longer treatment duration and the route of administration [6, 13]. Oral route is reported as at higher risk of nausea and vomiting and, in many cases, the switch to subcutaneous (SC) route may lead to improvement [13]. Conversely, other authors reported that a greater proportion of children and adults receiving parenteral MTX presented GI complaints compared to those treated with oral preparations [4, 6, 7]. This was confirmed in a multicenter cross-sectional study in which, at comparable median doses of MTX for the oral and the SC groups, a greater prevalence of nausea and vomiting was reported in the SC group (43% vs 29%), although without statistically significant difference [14].

Various strategies, such as folate supplementation or anti-emetics, and even switch to biological drugs, have been suggested to manage adverse GI effects of MTX in children and adults, but only few prospective studies have been published [11, 14, 15].

The rationale of using folic acid (FA) and LVF is related to their involvement in biosynthetic pathways independent of the dihydrofolate reductase enzyme inhibited by MTX. Therefore, the aim for using folates is to prevent possible effects such as megaloblastic anemia or cytopenia, and some studies showed that they reduce liver function abnormalities too [5, 15].

To date, few studies of folic acid use have been performed in children with JIA [16, 17]. One is a randomized double-blind placebo-controlled study involving 19 subjects showing that 1 mg/day of FA did not reduce MTX efficacy over a 12-week period, but effect on GI complaints was not analyzed [16]. Ravelli et al. published a retrospective study including 43 JIA patients presenting reduction of MTX-induced GI adverse effects from mean 1.09 to 0.29 episodes per patient-year after starting LVF supplementation [17].

Our study is the first prospective study demonstrating the long-lasting efficacy of LVF in reducing MTX-related GI complaints. All patients in our cohort were already taking LVF supplementation and the strategy of adding another dose 48 h before MTX proved effective, thus suggesting a possible reduced folate availability in these patients. In fact, several studies in adults showed that MTX-related adverse effects are associated with more severe folate deficiency [15, 18]. Furthermore, a lower concentration of both intracellular and whole blood folate isoforms was observed in children that had been treated with MTX and had experienced serious side effects, even many months after the drug was discontinued [19].

The results of our study, although with the limitations due to the small number of patients and the lack of a control group, may suggest some valuable considerations. Our data show that higher dosage of folates may be beneficial in patients with MTX-related GI complaints and also the route of administration may be important, as in our patients the strategy of giving LVF before MTX might have contributed to effectiveness. Indeed, the optimal folate regimen is still matter of debate and there is great variability among clinicians on prevention and treatment of MTX-related side effects [9, 20]. Therefore, further studies using LVF or FA before MTX since the start of the treatment should be performed to evaluate if this modality is effective also in preventing the development of adverse effects.

Another important result of the present study is that, in our patients, the doubling of the dose of LVF supplementation was not associated with changes in JIA disease activity measures such as the composite tool for scoring, JADAS, and the acute phase reactants, ESR and CRP.

In conclusion, our results suggest that in patients with JIA treated with MTX doubling the dose of LVF can be associated with a significant reduction of MTX-related side effects thus improving patients’ compliance and doesn’t affect disease activity.

## Data Availability

Please contact author for data request.

## Abbreviations

LVF: Levofolinic acid
MTX: Methotrexate
JIA: Juvenile Idiopathic Arthritis
GI: Gastrointestinal
FA: Folic Acid
ESR: Erythrocyte Sedimentation Rate
CRP: C Reactive Protein
ANA: Antinuclear Antibody
ERA: Enthesitis related Arthritis
SC: Subcutaneous
JADAS: Juvenile Arthritis Disease Activity Score
VAS: Visual Analogue Scale
SD: Standard Deviation
DMARD: Disease Modifying Antirheumatic Drug
